# Does nutritional education affect the nutritional status, growth, and development of children with pectus excavatum?

**DOI:** 10.1007/s00383-025-06045-3

**Published:** 2025-05-25

**Authors:** Meryem Kahrıman, Gözde Arıtıcı Çolak, Duygu Sağlam, Mustafa Yüksel, Sultan Aslanhan

**Affiliations:** 1 Faculty of Health Sciences, Department of Nutrition and Dietetics, Acibadem Mehmet Ali Aydinlar University, Istanbul, Turkey; 2 Chest Wall Deformities and Pectus Association, Istanbul, Turkey; 3 Faculty of Health Sciences, Department of Nutrition and Dietetics, Istanbul Sabahattin Zaim University, Istanbul, Turkey

**Keywords:** Pectus excavatum, Medical nutrition therapy, Education, Deformity

## Abstract

**Purpose:**

Pectus deformity can cause cardiovascular, gastrointestinal, and genitourinary anomalies depending on the severity of the deformity, and this can affect the nutritional status of patients. This study aimed to investigate the effect of nutritional education on nutritional status and anthropometric measurements in patients with moderate pectus excavatum (PE).

**Methods:**

This study included patients diagnosed with pectus disease between the ages of 9 and 16 years. A questionnaire was administered, which included items regarding demographic information, eating habits, and physical activity levels. All patients were provided with nutritional education, including healthy diet principles. Subsequently, anthropometric measurements, such as height and body weight, were recorded, and 24-h food recall was taken.

**Results:**

A total of 40 children [31 (77.5%) men, 9 (22.5%) women] diagnosed with PE and moderate deformity severity participated in the study. After the provision of nutritional education, the patients’ body weight, WHO height and body mass index for age, as well as intake of energy, protein, and fat significantly increased.

**Conclusion:**

This study showed that nutritional education can improve the nutritional status of patients with moderate PE as it leads to an increase in anthropometric measurements and food/nutrient intake. Therefore, it would be beneficial to refer patients to a dietician to implement longer-term and improved educational models.

## Introduction

Childhood is a period in which all bodily organs and physiological systems continue to grow and mature over time. Especially, 10–19-year-olds typically gain 20% of their ultimate adult height and 50% of their adult weight. Furthermore, the skeleton undergoes considerable remodeling, with an increase in bone mass of up to 40%. Nutrition and growth have an undeniably close association [1]. In this period, growth and development transform and substantially impact a person’s health in later life as well as the health of their future children [2].

Pectus deformity is a morphological pattern of chest wall deformity, the most prevalent types being pectus excavatum (PE) and pectus carinatum (PC). PE is the more common form compared to PC [3]. The incidence of PE is reportedly 1/400 in white male babies at birth, and it is five times more common in males than in females [4, 5]. These childhood deformities cause various degrees of psychological, orthopedic, and physiological disorders depending on the severity of malformation [6, 7]. PE and PC, which have been subjects of publications since the sixteenth century, were initially considered to cause aesthetic problems. In the early twentieth century, they were reported to also cause functional problems [8].

Although most patients with pectus deformities do not have life-threatening functional problems in the intrathoracic organs, cardiovascular, gastrointestinal, and genitourinary anomalies may occur in severe cases. Depending on the severity of these malformations, psychological, orthopedic and physiological disorders may occur. Especially in cases of PE, the most important of these disorders is shortness of breath. Moreover, the patients have restricted physical activity, develop skeletal and respiratory diseases and cardiac problems, and shortened life expectancy [9, 10]. In addition to all this, it is reported that pectus patients may exhibit the behavior of hiding their breasts because they are embarrassed by the appearance of their chest wall, therefore their self-esteem may be lower and anxiety and depressive symptoms may be common in these patients [11].

Moreover, pectus deformity can negatively affect patients’ nutritional status due to the occurrence of the aforementioned anomalies [9]. Previous studies have reported that 33% of children with malnutrition have PC [12, 13]. In a study conducted by Park et al. in Korea, the height, body weight, and body mass index (BMI) of patients with PE were significantly lower than those of the healthy population [14]. Kuru et al. found that in Turkiye, 32.5% and 26.4% of the children with PE and PC, respectively, were underweight [15]. These findings suggest that children with pectus deformity are at risk of malnutrition. However, it is unknown whether such malnutrition is due to inadequate energy and nutrient intake, differences in basal metabolic rate, or gastrointestinal anomalies. This study aimed to evaluate the effect of nutritional education based on healthy diet principles on anthropometric measurements and nutritional status in children with PE. The hypotheses of this study are as follows:

Hypothesis 1: Nutritional education in children with PE will increase body weight and anthropometric measurements such as height and BMI.

Hypothesis 2: Nutritional education in children with PE will improve their nutritional status by increasing their intake of nutrients, such as energy, protein, and fat.

## Materials and methods

### Study population

This study enrolled 55 children (40 males, 15 females) between the ages of 9 and 16 who were visited the Pectus Society and its clinic between July 2019 and February 2020. The inclusion criteria for the study were being diagnosed with pectus excavatum and being under 18 years of age. Participants who were over 18 years of age and had another disease diagnosis that could affect the respiratory system were excluded from the study. Only 40 patients diagnosed with PE and moderate deformity severity were included in the final analysis.

### Measurements

#### Questionnaire on sociodemographic information

A face-to-face interview with patients who agreed to participate in the study was conducted, and a questionnaire containing items regarding demographic information and nutritional habits was used to obtain data.

#### Anthropometric measurements

The researcher took anthropometric measurements at the beginning and end of the study. Body weight was measured in the morning, in the fasting state, wearing light clothing, and using a scale with a sensitivity of ± 0.1 kg. Meanwhile, height was measured using a 0.01 cm precision stadiometer with the patient in the standing position, with the feet side by side and head in the Frankfort plane (The ear canal and the lower border of the orbit-eye socket are in line and parallel to the ground). Hairpins, hats, and shoes that could affect the measurement were removed. The BMI was calculated as body mass (kg)/height (m^2^) [16]. Subsequently, the height-for-age and BMI-for-age percentages were determined using the WHO Anthro Plus program [17]. The patients were classified according to malnutrition status using the reference indicators of the Academy of Nutrition and Dietetics/American Society for Parenteral and Enteral Nutrition [18].

#### Food frequency questionnaire and 24-h food recall

At the beginning of the study, the patients’ dietary habits were evaluated using a food frequency questionnaire. In addition, 24-h food recalls were taken before and after the provision of nutritional education. This approach entails meticulous cataloging of every item, meal, and beverage consumed throughout the course of these 1 day, including a thorough description of the items consumed, their quantity (portion size), brand (if applicable), and manner of preparation (e.g., cooking method, fat addition, recipe, and ingredients). The portions of foods were determined using the Food and Nutrition Photo Catalog [19]. Meanwhile, the daily intake of nutrients and food groups was calculated using BeBis 7.2 (Nutrition Information Systems package program) (Stutgart, Germany, Turkish version) [20].

#### Nutritional education

Individualized training sessions (30–45 min) were provided to all patients by the same trained dietitian. The themes of nutritional education focused on the protein, energy, and fluid needs of each patient. For this purpose, the “Four-Leaf Clover Poster” created by the Hacettepe University Department of Nutrition and Dietetics in 1992 was used as a visual guide to explain the food groups. In addition, the patients’ nutritional status was evaluated, and nutritional recommendations were made according to the Turkish Nutrition Guide for this age group (milk and dairy products, meat products, fruits and vegetables, etc.) [21]. Their compliance with their individual recommendations was monitored by a researcher via telephone until their next follow-up (10 weeks).

#### Physical activity level

Physical activity status was assessed using a daily activity registration form. These forms were filled out by the researcher after speaking with the patient at both the first and second visits.

### Ethical consideration

The study was approved by the Acıbadem Mehmet Ali Aydınlar University Medical Research Board (approval number; 2021–23/08). As the patients were below 18 years old, parental consent for study participation was obtained.

### Statistical analysis

Statistical analyses were conducted using the NCSS (Number Cruncher Statistical System) 2020 (NCSS LLC, Kaysville, Utah, USA) and SPSS (Statistical Package for the Social Sciences) 2020 statistical software. Quantitative variables were expressed as mean, standard deviation, as well as median, min, and max values, whereas qualitative variables were expressed as frequency and percentage. A box plot and the Shapiro–Wilks test were used to gauge the normality of the data distribution. The paired samples *t*-test and Wilcoxon signed-rank test were employed to assess data with normal and non-normal distributions, respectively, in the quantitative in-group evaluations. The marginal homogeneity test was used to evaluate the variation of categorical data among the dependent groups. *p* < 0.05 was considered to indicate statistical significance (95% confidence interval). 

## Results

The study was completed with a total of 40 children (31 males [77.5%] and 9 females [22.5%]). The mean age of the children was 13.1 ± 1.75 (see Table 1).

**Table 1:** Descriptive data of the patients

		*n* (%)
Gender	Male	31 (77.5)
	Female	9 (22.5)
Age	9–13 years old	22 (55.0)
	14–16 years old	18 (45.0)
Age (year) ($\overline{X }\pm SD$)	13.1 $\pm 1.75$	

*SD* standard sample, $\overline{X } Sample mean$

The increases in height (*p* = 0.001) and body weight (*p* = 0.001) at the second visit compared with the first in the male patients were statistically significant. When the anthropometric measurements were evaluated with reference to the WHO growth charts, a significant increase in the BMI-for-age percentages (%) and z-scores was observed (*p* = 0.001) (Table 2).

**Table 2:** Changes in the anthropometric measurements of male patients provided with nutritional education

	First Visit		Second Visit		Difference ∆	*P*
	Avg ± SD	Median (min–max)	$\overline{X }$±SD	Median (min–max)		
Height (cm)	165.00 ± 10.92	166 (133–182)	165.98 ± 10.72	167 (133–182)	0.98 ± 1.10	^a^0.001**
Weight (kg)	45.07 ± 8.97	47 (28.2–65)	47.15 ± 9.27	47 (30.5–67)	2.08 ± 1.79	^a^0.001**
BMI (kg/m^2^)	16.42 ± 2.07	16.3 (10.3–20.7)	17.03 ± 2.19	17.1 (10.9–21.1)	0.61 ± 0.72	^a^0.001**
WHO BMI for age (%)	20.36 ± 20.98	12.1 (0.2–79.4)	28.56 ± 23.60	23.7 (0.3–73.5)	8.79 ± 10.62	^a^0.001**
WHO BMI fora ge (z-score)	− 1.45 ± 1.39	− 1.2 (− 6.4–0.8)	− 1.11 ± 1.39	− 0.9 (− 5.9–0.6)	0.37 ± 0.42	^a^0.001**
Activity factor	1.21 ± 0.10	1.18(1.1–1.5)	1.21 ± 0.10	1.18(1.1–1.5)	^***−***^	***1.000***

Bold italics results are statistically significant

*BMI* body mass index, *WHO* World Health Organization, $\overline{\text{X}}\text{ sample mean }$

^a^Paired samples *t*-test,

^**^P < 0.01

Similar to the male patients, significant increases in height (*P* = 0.001) and body weight (*P* = 0.001) were observed in the female patients. There were also significant increases in BMI-for-age percentages (%) as well as z-scores (*P* = 0.001) (Table 3).

**Table 3:** Changes in the anthropometric measurements of female patients provided with nutritional education

	First Visit		Second Visit		Difference ∆	*P*
	Avg ± SD	Median (min–max.)	$\overline{X }$±SD	Median (min–max.)		
Height (cm)	155.56 ± 7.72	158 (141–165)	156.44 ± 7.49	159 (141–165)	0.89 ± 1.05	^a^0.001**
Weight (kg)	37.61 ± 4.71	40 (30–42.5)	38.73 ± 4.07	40.6 (31–43)	1.12 ± 1.02	^a^0.001**
BMI (kg/m^2^)	15.48 ± 0.78	15.2 (14.2–16.8)	15.78 ± 0.55	15.6 (15.1–17)	0.30 ± 0.33	^a^0.001**
WHO BMI for age (%)	12.08 ± 10.01	11.9 (1.7–33.3)	14.92 ± 9.29	12.4 (5.2–28.7)	2.84 ± 3.81	^a^0.001**
WHO BMI for age (z-score)	− 1.32 ± 0.54	− 1.2 (− 2.1– − 0.4)	− 1.12 ± 0.42	− 1.2 (− 1.6– − 0.6)	0.20 ± 0.21	^a^0.001**
Activity factor	1.25 ± 0.10	1.18(1.1–1.5)	1.25 ± 0.10	1.18(1.1–1.5)	^***−***^	***1.000***

Bold italics results are statistically significant

*BMI*, body mass index; *WHO*, World Health Organization, $\overline{\text{X}}\text{ Sample mean }$

^a^Paired samples *t*-test

^**^P < 0.01

The patients’ malnutrition status before and after the provision of nutritional education showed a statistically significant difference (*P* = 0.002). In particular, the percentage of patients with mild malnutrition decreased from 45 to 27.5%, while the percentage of patients with normal nutrition increased from 35 to 57.5% (Table 4).

**Table 4:** Malnutrition classification before and after the provision of nutritional education

Malnutrition classification	First visit		Second visit		*P*
	*n*	%	*n*	%	
Normal	14	35%	23	57.5%	
Mild malnutrition	18	45%	11	27.5%	
Moderate malnutrition	5	12.5%	3	7.5%	^a^0.002^*^
Severe malnutrition	3	7.5	3	7.5%	

^a^Marginal homogeneity test

^*^P < 0.05

The increase in energy (kcal), protein (g), fat (g), carbohydrate (g), and fiber (g) intake in the second visit compared with the first was statistically significant (*p* < 0.01). The average increase in the consumption of bread (*p* < 0.01), milk (*p* < 0.05), and meat (*p* < 0.05) in the second visit compared with the first was statistically significant. In contrast, a significant decrease in the consumption of ready-to-drink packaged beverages was observed (*p* < 0.01) (Table 5).

**Table 5:** Changes in the nutrient intake of patients at the first and second visits

	First visit		Second Visit		Difference ∆	*p*
	Mean ± SD	Median (min–max.)	Mean ± SD	Median (min–max.)		
Energy (kcal)	1755.16 ± 224.41	1799.3 (1064–2151.8)	1839.70 ± 249.10	1910.6 (1087.3–2325.9)	84.55 ± 137.58	^***a***^0.001**
Protein (g)	76.12 ± 15.18	73.4 (40.4–110.1)	80.14 ± 13.59	77.9 (42.3–110.1)	4.02 ± 6.10	^***a***^0.001**
Protein %	17.75 ± 3.33	18 (12–25)	17.85 ± 3.17	17.5 (12–25)	0.1 ± 0.63	^***a***^***0.323***
Fat (g)	73.48 ± 16.74	72.2 (42–113)	75.40 ± 16.10	74.2 (42–113)	1.89 ± 3.43	^***a***^0.001**
Fat %	37.23 ± 7.70	36 (25–61)	36.65 ± 8.08	36 (25–61)	− 0.58 ± 1.48	^***a***^0.019*
Carbohydrate (g)	195.60 ± 49.32	198.3 (61.6–300.8)	208.64 ± 58.10	211.1 (61.6–292.7)	13.03 ± 23.48	^***a***^0.001**
Carbohydrate %	45.10 ± 9.15	47 (18–59)	45.55 ± 9.49	47 (18–59)	0.45 ± 1.72	^***a***^***0.107***
Fiber (g)	17.81 ± 6.97	16.2 (5.4–32.4)	19.07 ± 6.54	18.1 (5.4–32.4)	1.26 ± 2.83	^***a***^0.008**
Cholesterol (mg)	335.62 ± 119.97	325.1 (82.8–670)	368.24 ± 102.98	346.3 (153.3–670)	32.62 ± 62.80	^***a***^0.002**
Bread group (g)	233.75 ± 85.87	244 (10–459)	265.83 ± 101.51	296.5 (10–459)	32.08 ± 41.84	^***b***^0.001**
Fruit group (g)	261.80 ± 159.91	200 (0–700)	276.05 ± 151.54	243.5 (0–700)	14.25 ± 77.88	^***b***^***0.093***
Vegetable group (g)	78.38 ± 55.89	72.5 (0–232)	79.23 ± 55.91	81 (0–232)	0.85 ± 8.08	^***b***^***0.345***
Nut group (g)	32.85 ± 33.39	20 (0–120)	33.50 ± 33.11	20 (0–120)	0.65 ± 4.11	^***b***^***0.317***
Milk group (g)	466.68 ± 207.83	444 (141–950)	489.53 ± 194.12	455.5 (141–950)	22.85 ± 54.4	^***a***^0.011*
Ready-to-drink packaged beverage group (g)	327.40 ± 349.79	195.5 (0–1034)	302.08 ± 321.31	195.5 (0–1034)	− 25.33 ± 66.54	^***b***^0.007**
Fat group(g)	13.35 ± 8.94	14 (0–35)	13.58 ± 8.64	14 (0–35)	0.23 ± 1.69	^***a***^***0.404***
Sugar, chocolate (g)	23.10 ± 20.59	20 (0–95)	23.08 ± 20.49	20 (0–95)	− 0.03 ± 2.03	^***a***^***0.938***
Meat group (g)	79.13 ± 42.78	76.5 (0–200)	102.75 ± 86.58	82 (0–355)	− 23.63 ± 65.80	^***a***^0.029*

Bold italics results are statistically significant

^a^Paired samples *t*-test

^b^Wilcoxon signed-rank test

^*^P < 0.05

^**^P < 0.01

## Discussion

Adequate nutrition can be achieved with the intake of calories, proteins, vitamins, minerals, and trace elements necessary for maintaining life and sufficient growth [22]. The childhood period is characterized by continuation in growth and development as well as the occurrence of physical, social, and psychological changes. During this period, the nutritional habits of individuals are established, and habits that will continue into adulthood are acquired [23].

Pectus is a congenital anterior chest wall deformity of unknown etiology [24]. It usually requires treatment as it can cause decreased exercise tolerance, postural disorders, as well as respiratory, cardiovascular, gastrointestinal, and genitourinary anomalies [24–26]. Depending on the severity of these complications and deformities, patients may be at risk of nutritional deficiency and malnutrition [14, 15]. This situation is especially serious during childhood, when growth continues rapidly. Accordingly, this study aimed to evaluate the effects of nutritional education on anthropometric measurements and nutritional status in patients aged 9–16 years who were diagnosed with PE. The study was completed with 40 patients, of whom 77.5% were male and 22.5% were female. Previous literature has emphasized that pectus is more common in the male group [24], which explains why most of the study participants were male.

Only a few studies in the literature have reported that patients with pectus have lower body weight and height than their peers [12–15]. Although the incidence of malnutrition is common as the disease severity increases, there are no detailed studies explaining the reason for the low height and body weight in these patients. For example, a study conducted by Park et al. in Korea involving patients with PE reported that the height, body weight, and BMI of these patients were significantly lower than those of the healthy population [14]. Meanwhile, a study conducted in Turkiye showed that 32.5% and 26.4% of children with PE and PC, respectively, were underweight [15]. Similarly, our study found that 65% of the patients with PE had malnutrition and only 35% had a normal nutritional status before the training sessions. This finding supports the literature and suggests that patients with pectus are at risk of malnutrition. Malnutrition may lead to a worsening of disease prognosis and an increase in postoperative risks in cases requiring surgery, as previously shown in the literature [27].

Although there is no study in the literature evaluating the effects of nutritional education or nutrition intervention in children with pectus, it is emphasized that optimization of the intake of energy, macronutrients, trace elements, and other micronutrients is effective in the management of childhood respiratory diseases [28]. A study evaluating the effects of short-term nutritional intervention on body composition and disease prognosis in children with bronchiectasis and interstitial lung diseases reported that the intervention improved anthropometric measurements, such as body weight, BMI, height, tricep skinfold thickness, and mid-arm circumference [29]. In this study, the BMI-for-age z-scores of the male and female patients increased after the prevision of nutritional education. Furthermore, the rate of patients with malnutrition decreased, whereas the rate of patients with normal nutritional status increased. These findings support the literature and suggest that the provision of nutritional education based on age-specific nutritional recommendations in the national nutrition guide contributes to the improvement of the nutritional status of children with pectus. Considering the effects of this short-term intervention, establishing disease-specific nutritional recommendations for children with pectus may be an important step. A systematic review of 827 studies examining the effectiveness of nutritional education in children and adolescents concluded that education improved anthropometric measurements [30]. This finding and ours emphasize that nutritional education can be effective in inducing behavioral changes and reflecting these changes on nutritional status in patients.

At the end of the study, an increase in the patients’ daily intake of energy, macronutrients, and food groups with the nutritional intervention was observed. Although there is no study evaluating the effects of nutritional education on nutrient intake in children with pectus, some studies have been conducted on the healthy population. Yuliantini et al. [31] evaluated the effects of balanced nutritional education provided to primary school students using puzzles and found that their intake of energy, carbohydrate, and protein increased. Furthermore, the increased energy and macronutrient intake in our study explains the improvement in anthropometric measurements.

When the mean values of the food groups consumed by the patients daily were examined, it was observed that the daily intake could be increased in all groups, albeit limited. Consuming at least five servings of vegetables and fruits daily is recommended to reduce the risk of chronic diseases, such as diabetes, and cardiovascular diseases that may occur in adulthood [32]. At the end of this study, it was found that the average daily consumption of vegetables and fruits increased. However, the daily average was approximately two servings, which was below the recommended amount. Furthermore, the dietary habits of university students was evaluated, it was observed that individuals were able to increase their daily consumption of fruits, eggs, and fish [33]. Consumption of milk and milk products starts to decrease in adolescence; a previous study showed that daily milk consumption increased with a 5-week nutritional intervention [34]. An increase in milk and meat consumption was also observed among the participants. The findings in the literature and our study emphasize the benefits of nutritional intervention for children with pectus.

Another important effect of nutritional education is its effect on gastrointestinal symptoms, because gastrointestinal complications is commonly seen in children with pectus [24]. Although none of the study patients were diagnosed with gastrointestinal disease, it was observed that constipation and reflux were the most common complaints. However, these complaints were alleviated with short-term nutritional intervention (data not provided).

The secondary effect of pectus is psychosocial stress. This condition may cause restrictions in the daily physical activities of patients; make them less active, shy, and timid; and result in less contact with other people and peers as well as psychological changes, such as depression [35]. One of the most common complaints in patients with PE is decreased exercise capacity. Decreased cardiac and lung functions are the suspected causes of this condition [36]. Notably, the patients of this study were found to have sedentary lifestyle. Regular exercise has a very important contribution to children’s healthy growth and development as well as increased socialization and quality of life. Therefore, acquiring regular exercise habits is crucial in patients with pectus.

Our study demonstrates that the deficiencies in the growth and development of children with PE can be corrected in the short term by developing correct nutritional strategies. Although the surgical method employed to treat the disease plays a pivotal role in addressing malnutrition, increasing nutrient intake, and weight was found to be possible with the right nutritional intervention. However, our study has some limitations. First, majority of the study participants were male. However, as stated in the literature, pectus mostly affects males [24]. Although this situation is considered to be normal, it is also necessary to conduct studies involving a homogeneous sample. Second, the study included a relatively small sample size. This should not be ignored in the generalization of the study findings. Third, only nutritional education was provided to the patients. Although nutritional education is an important approach to creating behavioral change, nutritional interventions based on disease-specific recommendations should also be developed.

## Conclusion

In conclusion, the patients with pectus are at risk of malnutrition and this may pose a risk for comorbid conditions and complications. Even nutritional education based on healthy nutrition recommendations for the general population in the national nutrition guide has significantly improved patients’ body weight, nutritional status and dietary intake. The frequency of malnutrition among patients has decreased, and the number of patients with normal nutritional status has increased. Additionally, patient's energy, protein, carbohydrate and fiber intake increased. In this regard, developing specific nutritional recommendations for this disease and providing patient-based interventions will be an important step. Furthermore, it is believed that in the next period, increasing the nutritional knowledge of this patient group using visual, auditory, and written materials as well as the provision frequency of nutritional education will significantly contribute to their growth and development. 

## Data Availability

The data presented in this study are available on request from the corresponding author due to privacy.
